# The Impact of Enhanced Behavioral Health Services on Total Healthcare Costs Among US Employers: A Site-Level Analysis of 19 Cohort Studies

**DOI:** 10.36469/001c.138634

**Published:** 2025-06-20

**Authors:** Matt Hawrilenko, Casey Smolka, Emily Ward, RuthAnne Kavelaars, Millard Brown, Adam M. Chekroud

**Affiliations:** 1 Spring Health, New York, NY, USA; 2 Department of Psychiatry Yale University School of Medicine, New Haven, Connecticut, USA

**Keywords:** mental health, return on investment, medical claims, employee assistance program

## Abstract

**Background:** The return on investment (ROI) of mental health care is a critical metric in an era of cost-conscious healthcare decision-making. However, selective reporting of positive study results may inflate ROI estimates. **Objective:** To quantify the mean and variation in employer-level ROI outcomes for a comprehensive behavioral health benefit program. **Methods:** Data were obtained from 19 employer-specific studies conducted between May 2023 and December 2024. Sources included medical claims data spanning 12 months pre- and post-program launch, and program billing records of clinical and nonclinical costs. Studies were included if they were conducted by a single behavioral health benefit where the full sample of studies was known. The population included 19 US employers where employees and dependents received up to 12 free psychotherapy or medication management sessions. All studies used the same inclusion and exclusion criteria, retrospective matched cohort design, and difference-in-differences analysis. Data were abstracted following PRISMA guidelines. ROI was estimated using a difference-in-differences model to control for baseline medical spending and pooled using inverse variance weighting with a random effects structure. The primary outcome was the ROI multiple, defined as the ratio of gross per-member-per-month savings to total program spending. **Results:** The meta-analysis included 42 148 participants (14 645 program users and 27 503 matched controls) across a range of employer sizes and industries. The pooled ROI multiple was 2.3 (95% CI, 1.9-2.8), corresponding to net savings of 159permemberpermonth(95111-$207). Significant heterogeneity was observed (I² = 67.8%; t² = 0.646; *P* < .001). A sensitivity analysis including nonclinical costs yielded a pooled ROI of 1.8. **Conclusion:** This meta-analysis, the largest of its kind, demonstrates that a centralized behavioral health benefit can consistently generate net savings across varied employer settings. These findings provide robust evidence to support the adoption of comprehensive mental health programs as an effective strategy for reducing overall medical spending in employer-sponsored health plans.

## INTRODUCTION

In a healthcare environment increasingly focused on cost-efficiency, the return on investment (ROI) of mental health care has become a critical metric for organizations seeking to support individual well-being within budgetary constraints. A growing body of evidence suggests that fast access to high-quality mental health services through employee assistance programs (EAPs) may drive ROI by reducing total medical spending.^1–4^ However, the “file-drawer problem”—the selective reporting of only favorable findings—can artificially inflate ROI estimates, posing a significant challenge in evaluating the true ROI of EAP services.^5–7^

Compounding the issue of selective reporting is selective accounting. Even when results are reported, studies may exclude all mental health costs from the analysis,^8^ or selectively exclude the costs of mental health care obtained through the EAP but not the comparison group.^9^ For example, one series of studies reported reductions in health plan spending for a group of 4 employers but excluded the costs of mental health services obtained through the EAP (selective accounting)^9,10^; this study subsequently revealed EAP cost data only for a single employer whose health plan savings were substantially higher than previous reports (selective reporting).^11^ Thus, the absence of systematic evaluations across larger employer bases creates a critical knowledge gap that hinders evidence-based decision making about mental healthcare benefits.

The current study describes the range and variation of employer-level ROI outcomes for a comprehensive behavioral health benefit. Building on a previous pooled evaluation of seven employers,^4^ this study adds substantial new data to stratify outcomes across 19 employers. By including all available medical claims data, this study minimizes selection bias and aims to provide insights that accurately reflect real-world settings.

## METHODS

### Study Design

We conducted an individual and pooled random effects meta-analysis across 19 different financial ROI analyses of an employer-sponsored behavioral health benefit (Spring Health). Employer-level analyses were conducted between May 2023 and December 2024. Each employer contributed 2 consecutive years of data. The time window for claims data extraction encompassed services incurred from 12 months before program launch through 12 months after launch and paid through 15 months after launch. All services were incurred between November 2019 and May 2024. All studies used identical methods to a previous pooled evaluation.^4^ Briefly, a retrospective, matched cohort design was used to compare participants seeking behavioral health care (psychotherapy and/or medication management) through the behavioral health benefit to those who sought behavioral health care as usual via the health plan.

Participants were included if they had a behavioral health diagnosis at any time after program launch (index diagnosis), were insured by the health plan for at least 6 months prior and 1 month after the index diagnosis, aged 5 years and older, and did not have a transplant or end-stage renal disease. To cleanly identify the average treatment effect of the program, we required a clear binary exposure (program vs health plan). Participants who received care from both the program and the health plan during the study year (10% of program users) were therefore excluded. Allowing crossover between groups would violate the consistency and no-interference assumptions of the potential outcomes framework, since it becomes methodologically infeasible to disentangle which component of care drove changes in medical spending, and participants’ spending trajectories may depend on the order or timing of treatments received.^12^ Prior sensitivity analysis found that, given the small size of the group, such crossover impacts would be minimal.^13^ Because participants using the program were by definition starting a new episode of care, a washout period was implemented to mitigate survivorship bias in the control group, which may include individuals in long-term care or otherwise unresponsive to treatment. The washout period required all participants to have 6 consecutive months without treatment from a behavioral health specialist prior to their index diagnosis. Program group participants were identified as those having at least 1 psychotherapy or medication management session with the program. Nearest neighbor matching was used to match program users to a control cohort of nonusers.

Nearest-neighbor matching was used to match program users with health plan users at a 1:2 ratio. Participants were matched within each employer on mental health conditions (exact match on a collapsed category, where *International Classification of Diseases, Tenth Revision* [ICD-10] F codes were collapsed into 4 categories of mood, anxiety, substance use disorder, or other), log Department of Health and Human Services Hierarchical Condition Category [HHS-HCC] medical risk score (a caliper was applied to ensure standardized mean differences ≤0.1), age, sex, and date of first mental health diagnosis. Weights were used to adjust for participants who only received 1 match. Prior to analysis, matching variables were checked for balance across groups with a criterion of standardized mean difference ≤0.2.

Patients were followed the year prior to their first diagnosis in the study period (the index diagnosis) and up to 1 year following their index diagnosis. Risk scores were used from the index date (the first month of the post-phase) to capture new medical information occurring at the time of diagnosis.

Human subjects oversight was provided by the Yale University Institutional Review Board, which deemed this study “not human subjects” research and exempt from the need for informed consent (IRB protocol ID: 2000029276).

### Benefit Program

The Spring Health program is an employer-sponsored mental health benefit that provides up to 12 free psychotherapy sessions with a provider, up to 2 of which could be used for medication evaluation and treatment. Care beyond the employer-sponsored session limit was available as an in-network health plan benefit. The program uses evidence-based components including measurement-based care, unlimited care navigation sessions, and free access to a digital self-help app alongside video or in-person sessions with providers.

### Data Sources

*Medical claims data* were full medical claims incurred from 12 months prior through 12 months post-program launch, prepared as described previously (see **Supplementary Online Material**).

*Benefit program cost data* were obtained from the program’s billing records and separated into clinical care costs and non-clinical costs. The primary analysis focuses on the clinical care costs for psychotherapy and medication management, which are generalizable beyond the EAP to other mental health treatment settings. To err on the side of transparency and conservatism, a supplementary analysis also included non-clinical costs for services such as manager training and development, manager consultations, critical incident response, and access to a digital self-help app. Nonclinical costs of all health plan members–including those who did not engage in care with the program and were not included in the study–were allocated across the program group. Further detail is available in the **Supplementary Online Material**.

*Medical risk scores* were calculated using the HHS-HCC risk adjustment model, with factor weights from the 2019 platinum metal tier.^14^ The HHS-HCC risk score model accounts for over 150 health conditions and reflects risk for medical spending. A risk score of 1.0 represents average medical liability and scores scale proportionally; for example, a score of 2.0 represents twice average annual liability. Risk scores were calculated over the 12 months up to and including the date of index behavioral health diagnosis.

### Statistical Methods

Each individual ROI study used identical procedures to those described elsewhere.^13^ Program participants were matched at up to a 2:1 ratio to controls who sought care via the health plan, with weights applied when only 1 match was available. A weighted difference-in-differences model was fit using the *survey* package in R version 4.4.^15^ The difference-in-differences represents the association between program participation and changes in PMPM spending after the index diagnosis.

*Missing data.* Analyses were based on full medical claims extracts. Several insurers provided paid amounts rather than allowed amounts, and in these cases we imputed allowed amounts using the formula Allowed = Paid/0.8.^16^ Services that were incurred but not yet billed would not be included in the medical claims. The study relies on the assumption that patterning in unbilled claims is similar between groups.

### Pooled Analysis

Meta-analytic pooling was used to aggregate effects across the 19 studies using the R package meta. ROI multiples were calculated as the ratio of gross savings to total spending and then pooled using inverse variance weighting.^17^ A random-effects model was employed to test heterogeneity across employers. The null effect was defined as 1.0, the level where the costs of the program are fully offset by savings in medical claims, effectively rendering the benefit free to the employer.

## RESULTS

### Sample Characteristics

The sample included data from 19 employer-specific studies and a total of 42 148 participants (14 645 program users and 27 503 matched controls). Employer characteristics are shown in **Table 1**. Employers ranged in size from commercial employers with fewer than 5000 employees to “jumbo” employers with more than 35 000 employees. Employers represented diverse industries including technology, manufacturing, media, and health care. The average medical spending was $429 per member per month, and risk scores reflected mean annual liability 9% greater than the US population average, but with a wide range across employers (52.2%-163% population average spending). The sample also had a wide range of behavioral healthcare engagement in the year prior to the program (2.2%-12.3%, mean = 8.5%). Average employer-level utilization was 4.8 sessions.

**Table 1.** Employer Characteristics

**Characteristic**	**Overall (N=19)**
Industry, n (%)	
Education	1 (5.3)
Health care	6 (31.6)
Insurance	1 (5.3)
Manufacturing	4 (21.1)
Media	2 (10.5)
Retail	2 (10.5)
Technology	2 (10.5)
Transportation	1 (5.3)
Employer size category, n (%)	
3001-5000 employees	1 (5.3)
5001-15 000 employees	9 (47.4)
5001-35 000 employees	5 (26.3)
>35 000 employees	4 (21.1)
Baseline medical spending (PMPM, $)	
Mean (SD)	431 (92.3)
Median [min, max]	429 [272, 594]
HHS-HCC medical risk score	
Mean (SD)	1.09 (0.313)
Median [min, max]	1.14 [0.522, 1.63]
Pre-launch year behavioral health utilization (%)	
Mean (SD)	8.25 (3.31)
Median [min, max]	9.10 [2.20, 12.3]
Benefit program plan design, n (%)	
Bundled	4 (21.1)
Bundled + sponsored	3 (15.8)
Sponsored	12 (63.2)
No. (%) of sponsored sessions	
6	10 (52.6)
8	6 (31.6)
10	1 (5.3)
12	2 (10.5)
Average No. of therapy/medication sessions	
Mean (SD)	4.82 (0.745)
Median [min, max]	4.90 [3.66, 6.23]

Abbreviations: HHS-HCC, US Department of Health and Human Services Hierarchical Condition Category; PMPM, per member per month.

### Meta-Analytic Estimates

**Figure 1.**
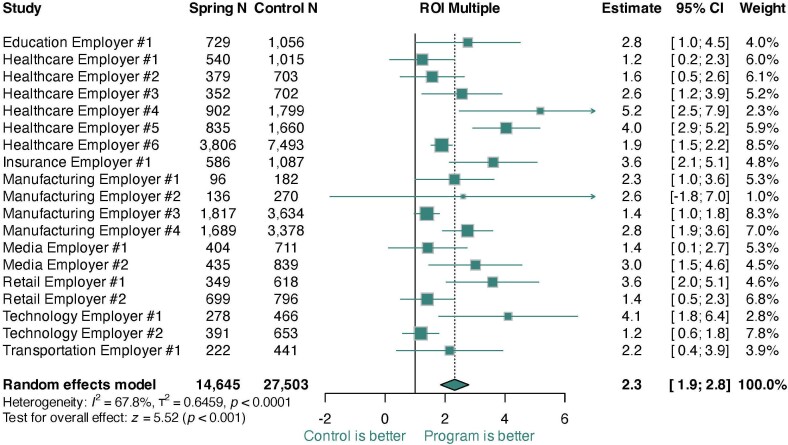
Meta-Analytic Results of ROI Studies Across 19 Employers Abbreviations: CI, confidence interval; ROI, return on investment.

The pooled ROI multiple across all studies was 2.3 (95% CI, 1.9-2.8), with all 19 employers experiencing net positive ROI (**Figure 1**). The ROI estimate corresponded to annual net savings of 14.3% (95% CI, 10.6%-18.1%; **Figures S1 and S2**) and gross savings of 25.2% (95% CI, 22.7%-27.6%; **Figure S3**). There was significant variation in ROI multiples across employers (*I^2^*= 67.8%; *t^2^*= 0.646, *P* < .001). However, when modeled as a percentage, heterogeneity was nonsignificant, suggesting that heterogeneity was driven by plan design or underlying population risk. Funnel analyses supported the notion that the study was not impacted by selection bias (**Figures S2 and S4**). A sensitivity analysis adding in costs for program elements unrelated to clinical care found similar but smaller effects (ROI = 1.8; **Figure S5**), with 17 of 19 employers showing net positive ROI and the remaining 2 not significantly different from cost offset.

## DISCUSSION

This study represents the largest ROI evaluation of a comprehensive behavioral health benefit program to date, leveraging data from 19 employers and 42 148 participants. The findings demonstrate consistently positive ROI across multiple industries with predominantly blue collar (eg, manufacturing) and white collar (eg, tech) job roles, medical risk ranging from 0.5× to 1.6× the national average, and varying propensity to use behavioral health care. Across all 19 studies, employers saved an average of 2.3 dollars on gross health plan spend for every dollar spent on program clinical care costs, corresponding to 14.3% net and 25.2% gross savings. This pattern of findings suggests that high-quality mental health care can consistently drive net medical savings, regardless of the heterogeneity in employee populations.

From a pragmatic perspective, these findings carry two main implications for employers. First, although the magnitude of savings varied across settings (ROI multiple range: 1.2-5.2), each of the 19 employers achieved a net positive ROI. Consequently, prospective adopters can reasonably anticipate net cost savings in their own settings, even amid variation in employee medical risk profiles. Second, the significant heterogeneity observed suggests that individuallevel characteristics moderate program impact, pointing to opportunities for more nuanced engagement strategies. Prior research has shown that employees with higher medical risk tend to drive larger reductions in total medical spending after engaging with behavioral health services. Employers can therefore amplify returns by using claims-based risk stratification to identify and proactively outreach to these higher-risk groups, tailoring communication and care navigation to optimize program uptake among those most likely to generate incremental savings. Such targeted engagement represents a straightforward, data-driven strategy to translate the overall positive ROI observed here into increased financial and clinical benefits.

The consistent findings across heterogeneous employer populations underscore the importance of understanding the active mechanisms driving cost savings. These mechanisms can broadly be split into 2 categories: return and investment. The return, corresponding to the numerator of the ROI multiple, is reduction in health plan spending. Previous research in a subset of the current sample found that savings were driven by reductions in physical health spending among participants with chronic physical health conditions,^13^ as mental health care drives physical health improvements both directly through biological pathways (eg, stress-cortisol-blood pressure^18,19^) and indirectly through improved health behaviors (eg, medication adherence and physical activity^20,21^). Even in populations that skew young and healthy, individuals selecting into mental health care have substantially higher rates of medical comorbidities,^3^ and their ensuing reductions in health plan spending can sustain a positive ROI across a wide array of employer settings.

Although the return in mental health care is reasonably well understood, its link to investment has not been well established. Critically, the efficiency of care may vary widely across implementations. For example, the present program has been shown to achieve stronger outcomes than evidence-based treatment alone^22–24^ in fewer sessions than comparable benefit programs.^9^ Unique programmatic aspects that may contribute to care efficiency stem from the program’s centralized system of mental healthcare delivery. All providers in the network operate within a single electronic health record platform, allowing case management, clinical supervision, and collaborative care teams to coordinate care in the same system with accurate and timely data. Moreover, data from every patient, provider, and interaction can be aggregated, analyzed, and leveraged to improve care delivery. Consistent with the data flywheel effect,^25^ the program’s outcomes improved as it scaled and collected more data, and in particular after the implementation of a patient-provider matching algorithm that leveraged data on each provider’s historical outcomes.^24^ Health systems that can improve the likelihood of success during a patient’s first care episode can increase care efficiency by reducing the likelihood of stalling in care or churning through multiple providers in search of the right fit.

Although the downstream reductions in medical spending observed here are likely achievable with other high-quality, evidence-based behavioral health interventions, the magnitude of net ROI may depend on program-specific efficiencies. The program evaluated in this study is unique in its combination of centralized care coordination and a scale-driven data feedback loop that has been associated with enhanced clinical outcomes in relatively few sessions.^24^ Other programs that deliver comparable clinical benefits—but require greater session volumes or lack real-time data-driven optimizations—may still reduce gross health plan spending, yet the balance between investment and return could differ. Future studies should examine how variations in treatment intensity, data infrastructure, and care-coordination features influence the cost-effectiveness of behavioral health benefits across diverse settings.

### Limitations and Future Directions

This study represents a real-world analysis at a scale beyond any prior studies of its kind. Because these 19 studies were non-randomized, ROI estimates depend on the assumption that matching adequately addressed biases to self-select between the program and care as usual via the health plan. If control group participants initiated care for unobserved and systematically different reasons than program participants—such as new or unmeasured physical health events—study estimates could still be biased. While we attempted to mitigate this bias through design (ie, a difference-in-differences analysis that adjusts for baseline spending) and matching choices (eg, using risk scores covering 150+ conditions from one year prior up to and including the month of the index event), we cannot fully eliminate it. Generalizability may be limited because the program itself did not vary across employers, limiting our ability to understand how different program components impact ROI. It would be beneficial to assess a wider degree of EAPs with differing program components; to that end, we hope the current study provides a model for other EAPs to consider when evaluating their ROI.

### Disclosures

All authors report being employed by, and holding equity in, Spring Health, the benefit program under study in this project.

## Supplementary Material

Online Supplementary Material
