# Supplementary material for: The Impact of Enhanced Behavioral Health Services on Total Healthcare Costs Among US Employers: A Site-Level Analysis of 19 Cohort Studies
Source: J Health Econ Outcomes Res. 2025 Jun 20;12(1):246–51. doi: 10.36469/001c.138634 (PMC12182909; doi:10.36469/001c.138634)
Supplement: Online Supplementary Material [file jheor_2025_12_1_138634_290163.pdf]

Journal of Health Economics and Outcomes Research

Online Supplementary Material

The Impact of Enhanced Behavioral Health Services on Total Healthcare Costs Among US Employers: A Site-Level Analysis of 19 Cohort Studies. *JHEOR*. 2025;12(1):246-251. doi:10.36469/jheor.2025.138634

Figure S1: Meta-Analysis of 19 Employer ROI Studies, Annual Net Savings (%)

Figure S2: Funnel Plot of Meta-Analysis Results, Annual Net Savings (%)

Figure S3: Meta-Analysis of 19 Employer ROI Studies, Annual Gross Savings (%)

Figure S4: Funnel Plot of Meta-Analysis Results, Direct Clinical Care ROI Multiple

Figure S5: Meta-Analysis of 19 Employer ROI Studies: EAP Costs, Inclusive of Non-Clinical Costs

This supplementary material has been provided by the authors to give readers additional information about their work.

This is an open-access article distributed under the terms of the Creative Commons Attribution 4.0 International License (CCBY-4.0). View this license's legal deed at http://creativecommons.org/licenses/by/4.0 and legal code at http://creativecommons.org/licenses/by/4.0/legalcode for more information.

Medical Claims Data Preparation

Allowed amounts were used to calculate medical spending using a minimum 3-month runout period (ie, claims incurred through 12 months post-launch used medical claims records through 15 months post-launch). When allowed amounts were not available (2 employers), paid amounts were converted to allowed amounts using the formula, Paid = Allowed / 0.8. Allowed amounts were truncated at $50 000 per claim and $100 000 per month.

Benefit Program Fee Structure

Clinical care costs were for mental health treatment sessions and incurred in a variety of fee structures (sessions billed to the employer, sessions prepaid by the employer regardless of whether they were used, sessions billed to the health plan, cancellation and no-show fees).

The benefit program has 2 main plan structures that include 4 types of billing. The first plan structure, called Direct Bill, bills employers per session for a set (maximum) number of sessions. The second plan structure, called Bundled, is a fixed per-employee-per-month (PEPM) payment regardless of utilization. In each plan structure, employers subsidize a certain number of sessions for employees, with additional sessions billed to the health plan as in-network providers.

Direct bill to employer: This is the total amount billed for all psychotherapy and medication management sessions, including no-show session fees.

Billed to health plan: This includes all Spring Health sessions that went beyond the number of sponsored sessions and were billed to the health plan.

Bundle fees: Bundle fees include the PEPM fees for all health plan members, including those who did not engage with care and were not included in the study.

Platform fees: Platform fees include the PEPM fees that provide access to Moments/Bloom digital health exercises, care navigation sessions, crisis support, and other services not included in the study. Platform fees were included for all health plan members, not just those included in the study. Although platform fees are not direct care costs, they were included in this study to err on the conservative side.

Return-on-Investment Calculation

The return on investment calculation was performed as follows:

Gross Savings = No. of program participants * Gross difference-in-differences * Average months post-index date

Total Program Costs = Direct bill + Billed to health plan + Bundle fees + Platform fees

ROI Multiple = Gross Savings / Total Program Costs

A return on investment = 1 represents a full cost offset of the benefit program, with values >1 representing net positive return on investment (ROI).

Figure S1. Meta-Analysis of 19 Employer ROI Studies, Annual Net Savings (%)

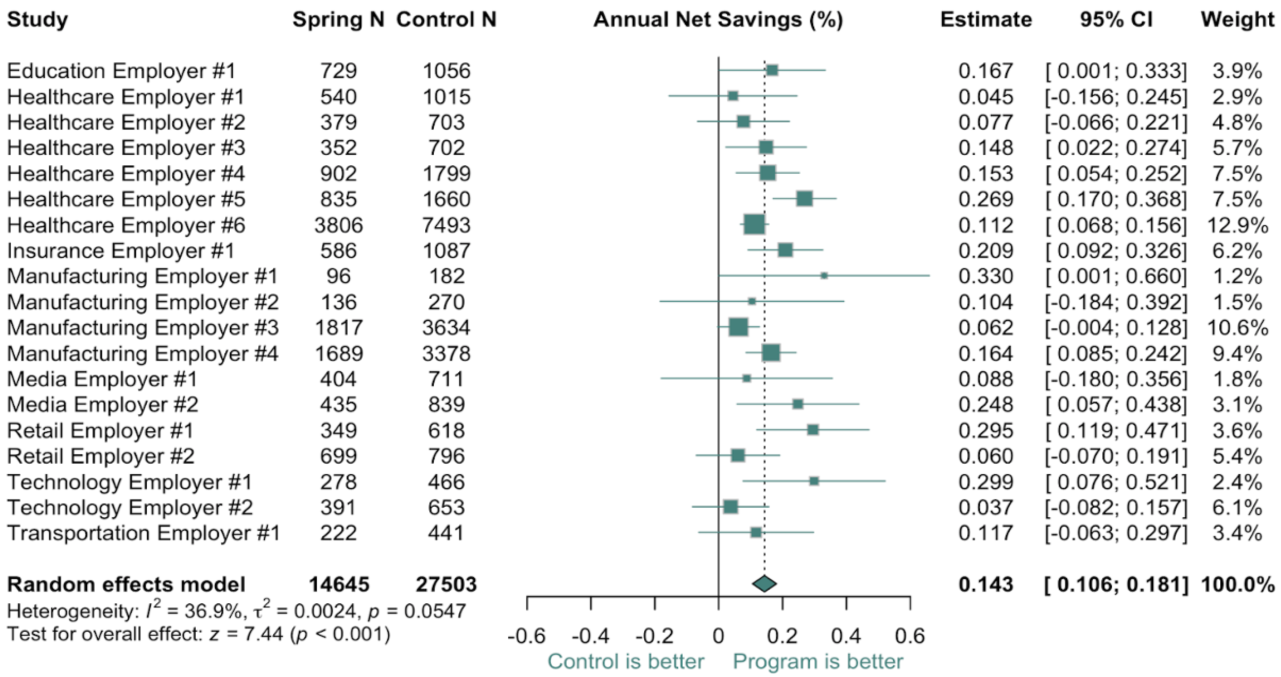

Figure S2. Funnel Plot of Meta-Analysis Results, Annual Net Savings (%)

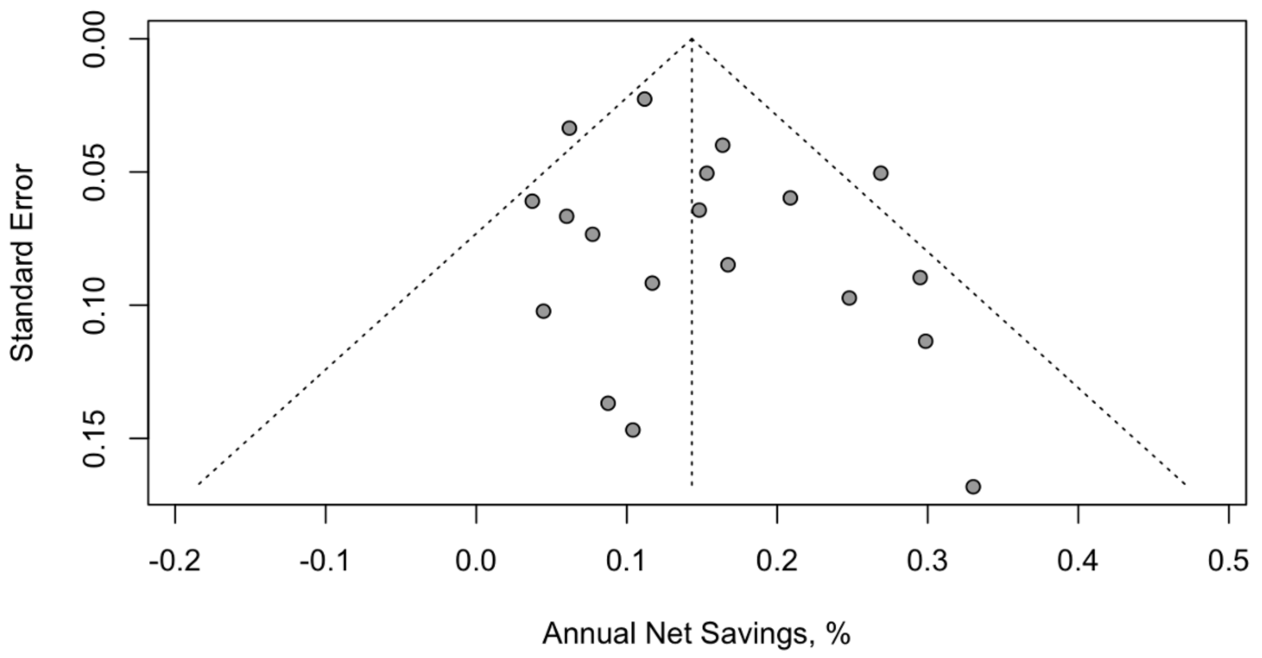

Each point represents one of the 19 employer-level ROI analyses. The vertical dashed line indicates the pooled net savings (14.3%), and the diagonal dashed lines delineate the expected 95% pseudo-confidence bounds under no small-study effects. The symmetric, triangular shape—wider at higher standard error and narrowing at lower standard error—supports the absence of selective-reporting bias.

Figure S3. Meta-Analysis of 19 Employer ROI Studies, Annual Gross Savings (%)

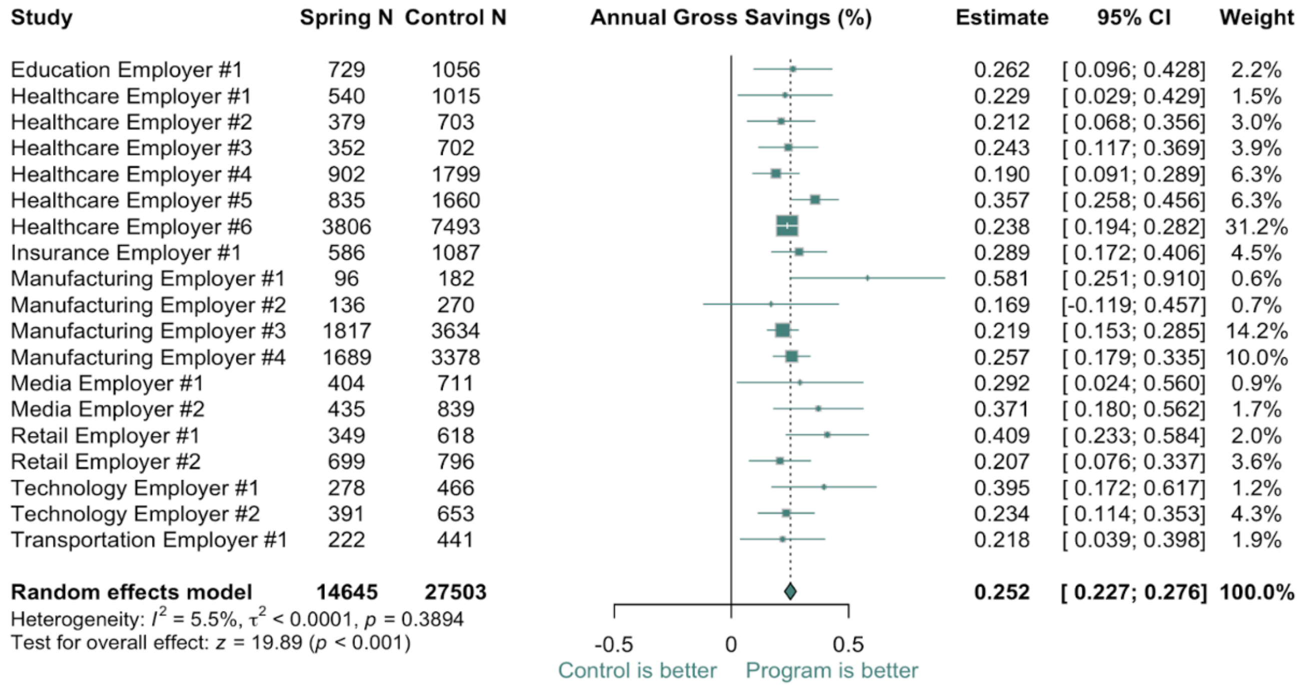

Abbreviations: CI, confidence interval; ROI, return on investment.

Figure S4. Funnel Plot of Meta-Analysis Results, Direct Clinical Care ROI Multiple

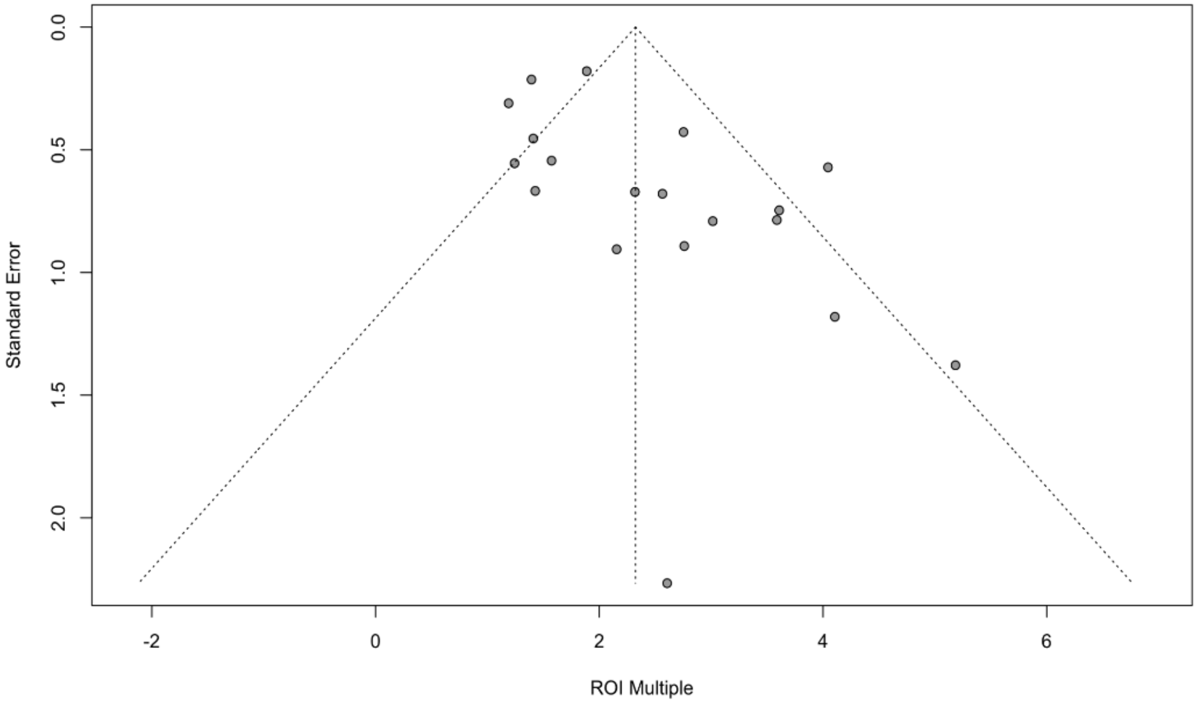

Abbreviations: ROI, return on investment.

Each point represents one of the 19 employer-level ROI analyses. The vertical dashed line indicates the pooled ROI multiple (2.3x), and the diagonal dashed lines delineate the expected 95% pseudo-confidence bounds under no small-study effects. The relative symmetry around the vertical line, wider at higher standard errors, supports the absence of selective-reporting bias.

Figure S5. Meta-Analysis of 19 Employer ROI Studies: EAP Costs, Inclusive of Nonclinical Costs

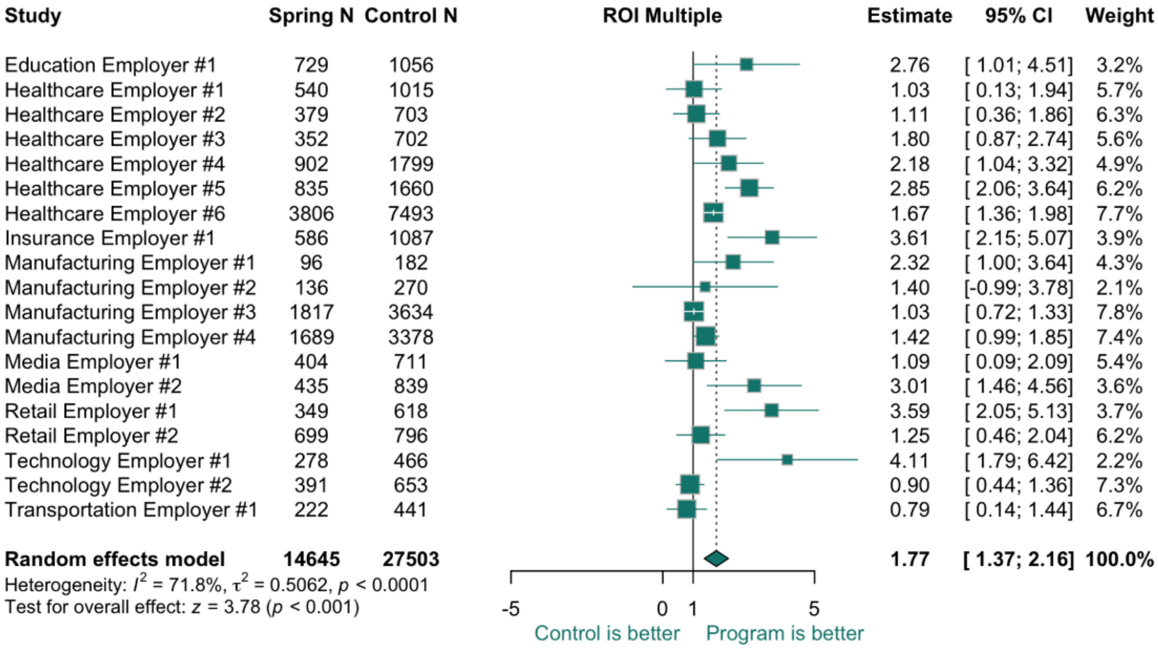

Abbreviations: CI, confidence interval; EAP, employee assistance program; ROI, return on investment.
